# Analysis of early haematological warning indicators for drug-resistant *Mycoplasma pneumoniae* infection

**DOI:** 10.3389/fped.2026.1854263

**Published:** 2026-10-21

**Authors:** Yuanyuan Yang, Tao Sun, Changyu Fu, Xiaofang Yan

**Affiliations:** Department of Pediatrics, The Fourth Affiliated Hospital of Soochow University, Suzhou, China

**Keywords:** haematologic tests, lymphocytes, macrolide resistance, monocytes, *Mycoplasma pneumoniae*

## Abstract

**Objective:**

This study aimed to investigate the haematological changes in children with *Mycoplasma pneumoniae* (MP) infection carrying the 2063/2064 mutation, providing a clinical reference for the early identification of drug-resistant MP.

**Methods:**

A retrospective study was conducted on 161 children diagnosed with MP infection via nucleic acid testing in our hospital in 2024, alongside 27 healthy controls. Based on the 2063/2064 mutation status, the MP group was divided into a mutation-undetected group (*n* = 27) and a mutation-detected group (*n* = 134). Haematological parameters and inflammatory indices at diagnosis were compared. Dynamic changes between the initial visit and diagnosis were analysed in 64 mutation-detected patients with complete paired data. Additional analyses included bootstrap resampling, *post-hoc* power assessment, exploratory ROC analysis, logistic regression and hospitalisation-stratified sensitivity analysis.

**Results:**

No significant differences were observed between the mutation-detected and mutation-undetected groups in clinical characteristics or haematological and inflammatory indices at diagnosis (all *p* > 0.05). Among the 64 mutation-detected patients with complete paired measurements, MLR decreased from 0.32 (0.24, 0.48) at the initial visit to 0.27 (0.19, 0.37) at diagnosis (*p* = 0.004). In the hospitalisation-stratified analysis, this decline was significant among hospitalised patients but not among non-hospitalised patients. Exploratory ROC analysis showed limited discriminatory performance for both the directional MLR change (AUC = 0.508, 95% CI 0.344–0.672) and its absolute magnitude (AUC = 0.523, 95% CI 0.349–0.699).

**Conclusion:**

Static haematological parameters and inflammatory indices did not distinguish mutation-detected from mutation-undetected MP infection. Although MLR decreased over time in the mutation-detected subgroup, ROC and stratified analyses showed that this change had limited discriminatory performance when used alone and may have been influenced by hospitalisation-related clinical factors. Dynamic MLR change should therefore be considered an exploratory marker requiring prospective validation.

## 1 Introduction

*Mycoplasma pneumoniae* (MP) infection is an important pathogen of community-acquired pneumonia in preschool and school-aged children. Studies show that MP accounts for approximately 10%–40% of the pathogens in community-acquired pneumonia in children, with a periodic epidemic every 3–7 years. In epidemic years, the infection rate can exceed 40% (1, 2). Tissue and cellular damage and necrosis caused by its unique molecular mechanisms, such as the P1 protein mediating *Mycoplasma* adhesion to host cells (3) and lipid-associated membrane proteins rich on the membrane surface that induce host production of pro-inflammatory cytokines, are key factors in its pathogenicity. Additionally, MP can induce the body to produce specific antibodies, and various cytokines affect the host immune system, leading to disordered humoral and cellular immunity and resulting in multi-system organ damage (4). Children with refractory *Mycoplasma* pneumonia associated with macrolide resistance have significantly higher fever duration and various complications, such as large amounts of pleural effusion, necrotising pneumonia and various laboratory indicators and imaging changes, compared with non-resistant MP infections (5–7), causing great distress and cost to the child, family and society. Currently, the main mechanism of MP resistance is considered to be point mutations in the V region of the 23S ribosomal RNA (rRNA) gene (positions 2063/2064) of MP, which reduces the affinity of macrolide antibiotics for *Mycoplasma* and decreases drug sensitivity (6, 8). This leads to prolonged respiratory and extrapulmonary symptoms in children in clinical settings, worsening the condition. These children experience persistent fever and cough, frequent medical visits, increasing their suffering and medical resource consumption. It is particularly important to identify these children early in outpatient settings, apply antibiotics rationally early and reduce severe complications and multiple hospital visits.

Blood cell analysis is the most easily accepted and least invasive examination method in outpatient settings. Therefore, determining whether it yields haematological clues for early resistant MP infection has become a matter of clinical urgency. Existing studies mainly focus on haematological changes in severe *Mycoplasma* pneumonia but lack a systematic analysis of early differentiation indicators between resistant and sensitive strains. This trial aims to explore how to identify resistant MP infection early through haematological and comprehensive inflammatory indices, using a retrospective case analysis approach. By dynamically monitoring changes in blood cell counts and inflammatory indices in children infected with macrolide-resistant MP (MRMP), this study focuses on analysing the evolution trend of the monocyte-to-lymphocyte ratio (MLR), providing a new perspective for early identification of resistant infections and aiming to provide a basis for the early rational application of antimicrobial drugs in clinical practice and reduction of severe complications.

## 2 Data and methods

### 2.1 General information

#### 2.1.1 Inclusion and exclusion criteria

Inclusion criteria for the MP infection group were as follows: (1) visited the hospital between January 2024 and December 2024; (2) respiratory pathogen nucleic acid testing indicated a positive MP, and the 2063/2064 mutation site was simultaneously detected; (3) aged ≤14 years; (4) the clinical data are complete.

Inclusion criteria for the normal control group included (1) randomly selected children who underwent physical examination or provocation tests during the same period, (2) aged ≤14 years and (3) no infection symptoms and negative nucleic acid of the pathogen.

Exclusion criteria included (1) co-infection with other respiratory pathogens and (2) missing clinical or experimental data.

#### 2.1.2 Grouping criteria

In this study, the participants were divided into the normal control group (*n* = 27) and the MP infection group (*n* = 161). Based on the detection results of the 2063/2064 mutation site, the MP infection group was further divided into (1) the group without detected mutation sites (i.e., the sensitive group, *n* = 27) and (2) the mutation site detection group (i.e., the study group/drug-resistant group, *n* = 134).

#### 2.1.3 Sample size estimation and reliability

The sample size for this study was determined using power analysis. Referring to the mean and standard deviation of the MLR of the two groups in the previous pilot data, values of *α* = 0.05 and 1 − *β* = 0.8 were set. Specifically, the pilot data yielded a mean MLR of 0.35 (SD = 0.18) in the sensitive group and 0.27 (SD = 0.14) in the resistant group, corresponding to an effect size of approximately 0.51 (Cohen's *d*). The calculation results show that to achieve statistical differences, the sample size of each group must be ≥25 cases. In this study, the sample sizes of both the study group (*n* = 134) and the sensitive group (*n* = 27) exceeded this minimum value, meeting the sample size requirements for statistical analysis.

#### 2.1.4 Ethics statement

This study was approved by the hospital's medical ethics committee.

### 2.2 Clinical methods

#### 2.2.1 Data sources and quality control

The clinical and laboratory test data of the children in this study were all derived from the hospital's electronic medical record system and the health records of Suzhou City. To ensure the accuracy and reliability of the data, all clinical information and laboratory indicators were independently extracted by two researchers, and quality control was performed through double-person entry and cross-checking to guarantee the accuracy of the data. The baseline information collected includes general data, such as gender and age.

#### 2.2.2 Experimental detection method

At the child's first visit and diagnosis, peripheral blood or peripheral venous blood was collected in the hospital's clinical laboratory. As the nature of the test was routine infection screening, the blood collection was not performed in a fasting state. Among the tests, the specific count values of white blood cell (WBC) count, neutrophil count, lymphocyte count and monocyte count were all determined by flow cytometry using the automated haematology analyser and its matching reagents. The level of hypersensitive C-reactive protein (hs-CRP) was determined by turbidimetry using the chemistry analyser and its corresponding reagents.

### 2.3 Observation indicators and definitions

#### 2.3.1 Supplementary explanation of time points

This study dynamically monitored the indicators at the following two key time points: (i) first visit—defined as the time point when the child first seeks medical attention after the onset of respiratory-related symptoms; (ii) at the time of diagnosis—defined as the time point when the nucleic acid test results of MP and the test results of the 23S rRNA mutation site are officially reported.

#### 2.3.2 Specific observation indicators

At the above two time points, the following indicators were closely observed: (i) blood cell analysis count values, including total WBC count, neutrophil count, lymphocyte count and monocyte count; (ii) an inflammatory indicator—hs-CRP protein count value; (iii) comprehensive inflammatory indices—neutrophil-to-lymphocyte ratio (NLR), MLR and systemic immune-inflammatory index (SIRI); (iv) clinical outcome indicators—the number of visits, blood samples, average duration of fever before diagnosis and hospitalisation of the child patient.

### 2.4 Statistical analysis

Statistical analysis was conducted using SPSS 25.0. Normality was assessed using the Shapiro–Wilk or Kolmogorov–Smirnov test. Non-normally distributed continuous variables are presented as median (interquartile range) and were compared between independent groups using the Mann–Whitney *U* test (*Z* statistic). Paired within-group comparisons between the initial visit and diagnosis were performed using the Wilcoxon signed-rank test (*Z* statistic). Categorical variables are presented as *n* (%) and were compared using the chi-square test (*χ*^2^ statistic). To evaluate the robustness of the paired MLR change in the mutation-detected group, bootstrap resampling with 20,000 iterations and replacement was performed, and the bootstrap-estimated standard error and 95% confidence interval were calculated. *Post-hoc* power for the inflammatory indices was estimated from r = Z/√*N* using a two-sided *α* of 0.05. Exploratory ROC analyses were performed for MLR1, defined as MLR at diagnosis minus MLR at the initial visit, and MLR2, defined as the absolute value of this difference; AUC with 95% CI, optimal cut-off, sensitivity and specificity were calculated. Binary logistic regression was used to assess the association of age and MLR at diagnosis with mutation-detected status. A hospitalisation-stratified sensitivity analysis was conducted among the 64 mutation-detected patients with paired MLR measurements, and an exploratory multivariable analysis incorporating sex, age, hospitalisation status, fever duration before diagnosis, ΔMLR and ΔCRP was also performed. All tests were two-sided, and *p* < 0.05 was considered statistically significant.

## 3 Results

### 3.1 Comparison of general characteristics and haematological parameters between *Mycoplasma pneumoniae* infection group and normal control group

Regarding baseline characteristics, there was no significant difference in gender distribution between the two groups (*p* = 0.721), and the median age in the MP infection group was significantly lower than that in the normal control group (7 vs. 9 years, *p* = 0.001). Compared with the normal control group, the MP infection group showed significant differences in haematological parameters, including WBC count, neutrophils, lymphocytes, monocytes and hs-CRP (all *p* < 0.05), which were consistent with clinical manifestations of infection. Regarding the interval from the first medical visit to diagnosis, the median time was 7.05 days (interquartile range: 4–8 days) in the mutation-detected group and 6.15 days (interquartile range: 5–7 days) in the mutation-undetected group. However, this difference was not statistically significant (*p* > 0.05) (Table 1).

**Table 1:** Comparison of hematological parameters and hs-CRP between *Mycoplasma pneumoniae* infection group and normal control group.

Observation index	Normal control group (*n* = 27)	Mycoplasma infection group (*n* = 161)	*χ*^2^/*Z*	*P*
Gender			0.13	0.721
Male	13	84		
Female	14	77		
Age (years)	9 (7, 11)	7 (5, 9)	−3.29	0.001
White Blood Cell Count (10^9^/L)	6.19 (4.86, 7.32)	7.39 (5.75, 9.02)	−2.80	0.005
Neutrophil Count (10^9^/L)	2.87 (2.18, 3.57)	4.57 (3.40, 5.93)	−4.71	<0.001
Lymphocyte Count (10^9^/L)	2.50 (2.06, 2.88)	1.85 (1.56, 2.61)	−2.87	0.004
Monocyte Count (10^9^/L)	0.33 (0.29, 0.37)	0.57 (0.42, 0.74)	−5.38	<0.001
High-sensitivity CRP (mg/L)	0.28 (0, 1.10)	9.38 (3.86, 18.92)	−7.21	<0.001

Data are expressed as median (interquartile range) [M (P25, P75)] for continuous variables or as frequency and percentage [*n* (%)] for categorical variables. Between-group comparisons for continuous variables were performed using the Mann–Whitney *U* test (*Z* statistic); between-group comparisons for categorical variables (e.g., Gender) were performed using the Chi-square test (*χ*^2^ statistic). The column header “*χ*^2^/*Z*” reflects the corresponding statistic for each row.

### 3.2 Comparison of general data between mutation-detected and mutation-undetected groups within the *Mycoplasma pneumoniae* infection cohort

There were no significant differences between the two groups regarding gender, age at infection, number of medical visits, number of blood collections, duration of fever at diagnosis, hospitalisation rate or time to confirmation (all *p* > 0.05) (Table 2).

**Table 2:** Comparison of general information between the mutation-undetected and mutation-detected groups in *Mycoplasma pneumoniae* infection patients.

Observation index	Mutation-undetected group (*n* = 27)	Mutation-detected group (*n* = 134)	*χ*^2^/*Z*	*P*
Gender			1.52	0.200
Male	11	72		
Female	16	62		
Age (years)	7 (5, 8)	7.0 (5.0, 9.25)	−1.09	0.274
Number of Visits	2 (1, 3)	2 (1, 3)	−0.48	0.633
Number of Blood Collections	2 (1, 2)	2 (1, 2)	−0.27	0.789
Fever Duration (days)	4 (1.5, 7)	4 (3, 6)	−0.01	0.991
Hospitalization Required	Yes	Yes	0.51	0.476
Time to Confirmation (days)	6 (5, 7)	6 (4, 8)	−0.63	0.532

Data are expressed as median (interquartile range) [M (P25, P75)] for continuous variables or as frequency and percentage [*n* (%)] for categorical variables. Between-group comparisons for continuous variables were performed using the Mann–Whitney *U* test (*Z* statistic); between-group comparisons for categorical variables were performed using the Chi-square test (*χ*^2^ statistic). The column header “*χ*^2^/*Z*” reflects the corresponding statistic reported for each row.

### 3.3 Comparison of haematological and inflammatory indices between mutation-detected and mutation-undetected groups at diagnosis

At the time of diagnosis, no significant differences were observed between the mutation-detected and mutation-undetected groups in haematological parameters—including WBC count, lymphocyte count, neutrophil count and monocyte count—or in inflammatory indices, such as the NLR, MLR, SIRI or hs-CRP (all *p* > 0.05) (Table 3).

**Table 3:** Comparison of hematological analysis and inflammatory indices between the mutation-detected and mutation-undetected groups in *Mycoplasma pneumoniae* infection.

Group	Not detected group	Detected group	*Z* value/*χ*^2^	*P* value
White Blood Cell Count (10^9^/L)	7.03 (5.72, 9.85)	7.46 (5.74, 8.98)	−0.63	0.528
Neutrophils (10^9^/L)	4.7 (3.39, 6.31)	4.57 (3.4, 5.93)	−0.48	0.628
Lymphocytes (10^9^/L)	1.78 (1.48, 2.46)	1.87 (1.56, 2.62)	−0.24	0.814
Monocytes (10^9^/L)	0.61 (0.38, 0.87)	0.56 (0.42, 0.73)	−0.67	0.506
NLR	2.56 (1.39, 3.69)	2.21 (1.49, 3.37)	−0.64	0.521
MLR	0.28 (0.2, 0.49)	0.27 (0.19, 0.38)	−0.6	0.552
SIRI	1.37 (0.69, 2.92)	1.24 (0.77, 1.99)	−0.82	0.414
hs-CRP (mg/L)	5.93 (1.32, 21.25)	9.55 (3.93, 18.92)	−1.01	0.313

### 3.4 Dynamic changes of indicators in the mutation-detected group (study group) at different time points

The MLR at diagnosis was significantly lower [0.27 (0.19, 0.37)] than that at the initial visit [0.32 (0.24, 0.48); *Z* = −2.85, *p* = 0.004]. No significant paired changes were observed in WBC count, lymphocyte count, neutrophil count, monocyte count, NLR, SIRI or hs-CRP (all *p* > 0.05) (Table 4).

**Table 4:** Intragroup comparison of the *Mycoplasma pneumoniae* infection mutation-detected group (initial visit vs. diagnosis).

Group	Initial visit	Diagnosis	*Z*	*P*
White Blood Cell Count (10^9^/L)	7.12 (6.10, 8.35)	7.29 (5.69, 9.03)	−0.04	0.971
Neutrophil Count (10^9^/L)	4.63 (3.97, 5.71)	4.94 (3.29, 5.64)	−0.78	0.438
Lymphocyte Count (10^9^/L)	1.71 (1.42, 2.25)	1.73 (1.48, 2.52)	−1.25	0.211
Monocyte Count (10^9^/L)	0.57 (0.47, 0.75)	0.54 (0.42, 0.75)	−1.43	0.151
NLR	2.71 (1.84, 3.46)	2.42 (1.65, 3.42)	−1.42	0.155
MLR	0.32 (0.24, 0.48)	0.27 (0.19, 0.37)	−2.85	0.004
SIRI	1.41 (0.95, 2.67)	1.31 (0.79, 1.86)	−1.90	0.136
hs-CRP (mg/L)	12.65 (6.72, 113.99)	9.84 (4.76, 19.60)	−0.47	0.640

Data are expressed as median (interquartile range) [M (P25, P75)]. Paired within-group comparisons (Initial Visit vs. Diagnosis) were performed using the Wilcoxon Signed-Rank Test (*Z* statistic), as these data represent longitudinal paired measurements from the same patients at two time points. The column header “*Z*” reports the Wilcoxon *Z* statistic.

### 3.5 Dynamic changes of indicators in the mutation-undetected group (sensitive group) at different time points

No significant differences were observed in WBC count, lymphocyte count, neutrophil count, monocyte count, NLR, MLR, SIRI or hs-CRP at the time of diagnosis (all *p* > 0.05) (Table 5).

**Table 5:** Intragroup comparison of the *Mycoplasma pneumoniae* infection mutation-undetected group (initial visit vs. diagnosis).

Group	Initial visit	Diagnosis	*Z*	*P*
White Blood Cell Count (10^9^/L)	7.9 (5.84, 12.6)	7.9 (6.73, 11.32)	−0.64	0.523
Neutrophil Count (10^9^/L)	4.23 (3.25, 9.73)	4.69 (3.38, 7.92)	−0.73	0.463
Lymphocyte Count (10^9^/L)	2.1 (1.67, 2.72)	2.49 (1.67, 3.04)	−0.33	0.740
Monocyte Count (10^9^/L)	0.68 (0.53, 0.93)	0.56 (0.4, 0.7)	−1.87	0.061
NLR	2.07 (1.45, 4.94)	1.5 (1.32, 4.73)	−0.03	0.981
MLR	0.35 (0.2, 0.49)	0.21 (0.18, 0.28)	−1.21	0.227
SIRI	1.15 (0.91, 4.70)	1.0 (0.72, 1.36)	−0.78	0.435
hs-CRP (mg/L)	5.0 (0, 11.15)	4.36 (0.98, 18.75)	−1.03	0.301

Data are expressed as median (interquartile range) [M (P25, P75)]. Paired within-group comparisons (Initial Visit vs. Diagnosis) were performed using the Wilcoxon Signed-Rank Test (*Z* statistic), as these data represent longitudinal paired measurements from the same patients at two time points. The column header “*Z*” reports the Wilcoxon *Z* statistic.

### 3.6 Bootstrap and *post-hoc* power analyses

Bootstrap resampling with 20,000 iterations yielded an estimated standard error of 0.0291 and a 95% confidence interval of −0.0988 to 0.0155 for the paired difference. Because the interval included zero, the bootstrap analysis did not independently confirm a non-zero mean paired difference. *Post-hoc* power was 0.11 for NLR, 0.09 for MLR, 0.14 for SIRI and approximately 0.17 for hs-CRP, all substantially below 0.80, indicating that the non-significant between-group findings may have been affected by insufficient statistical power (Supplementary Table S1).

### 3.7 Exploratory ROC analysis of MLR changes

For MLR1, the AUC was 0.508 (95% CI 0.344–0.672); the optimal cut-off was ≤−0.040, with a sensitivity of 57.8% and a specificity of 58.8%. For MLR2, the AUC was 0.523 (95% CI 0.349–0.699); the optimal cut-off was ≤0.220, with a sensitivity of 85.9% and a specificity of 29.4%. Both AUC values were close to 0.50, indicating limited discriminatory performance when MLR change was used alone (Supplementary Figure S1).

### 3.8 Association of age and MLR with mutation-detected status

In a binary logistic regression model with mutation-detected status as the dependent variable, neither age (B = −0.071, OR = 0.931, 95% CI 0.804–1.078, *p* = 0.340) nor MLR at diagnosis (B = 0.238, OR = 1.269, 95% CI 0.421–3.827, *p* = 0.673) was independently associated with mutation status (Supplementary Table S2). A resistance-status model incorporating ΔMLR and the full set of proposed covariates could not be estimated reliably because complete paired measurements required to calculate ΔMLR were not sufficiently available in the mutation-undetected group. Therefore, the reported model evaluates static MLR at diagnosis after adjustment for age and does not establish an independent association between ΔMLR and mutation status.

### 3.9 Hospitalisation-stratified and exploratory multivariable analyses

Among the 64 mutation-detected patients with complete paired MLR measurements, 49 were hospitalised and 15 were not hospitalised. In the hospitalised subgroup, MLR decreased from 0.35 (0.24–0.47) at the initial visit to 0.30 (0.19–0.34) at diagnosis (*Z* = −2.92, *p* = 0.004). In the non-hospitalised subgroup, MLR changed from 0.39 (0.23–0.52) to 0.37 (0.23–0.47), without a statistically significant difference (*Z* = −0.70, *p* = 0.480) (Supplementary Table S3).

In the exploratory multivariable analysis, none of the included variables reached statistical significance: sex, OR = 0.327 (95% CI 0.091–1.176, *p* = 0.087); age, OR = 1.103 (95% CI 0.888–1.370, *p* = 0.374); hospitalisation status, OR = 0.600 (95% CI 0.162–2.215, *p* = 0.443); ΔMLR, OR = 8.525 (95% CI 0.360–201.918, *p* = 0.180); ΔCRP, OR = 0.988 (95% CI 0.960–1.016, *p* = 0.388); and fever duration before diagnosis, OR = 1.174 (95% CI 0.933–1.478, *p* = 0.171) (Supplementary Table S4).

## 4 Discussion

This study examined whether routinely available haematological indices could help identify MP infections carrying the 2063/2064 mutation. At diagnosis, WBC subsets, hs-CRP, NLR, MLR and SIRI did not differ between the mutation-detected and mutation-undetected groups. Among mutation-detected patients with paired measurements, MLR decreased between the initial visit and diagnosis. However, the bootstrap, ROC, regression and hospitalisation-stratified analyses showed that the magnitude, discriminatory performance and specificity of this change were limited. The main finding is therefore not that MLR can diagnose resistance, but that a modest temporal change may occur in a clinically heterogeneous subgroup.

Compared with healthy controls, children with MP infection showed the expected changes in WBC subsets and hs-CRP, consistent with previous paediatric studies (9, 10). MP adherence and epithelial injury trigger innate and adaptive immune responses, including neutrophil recruitment, cytokine release and lymphocyte activation (11–14). These responses explain why routine blood tests can support the recognition of infection. Their relationship with macrolide-resistance mutations is less direct. Earlier studies of resistant or severe MP pneumonia have reported inconsistent differences in WBC, neutrophils, lymphocytes and CRP (15–19). In our cohort, these markers distinguished infected children from healthy controls but not mutation-detected from mutation-undetected infection. A marker of systemic inflammation may therefore identify infection without reflecting the molecular mechanism responsible for reduced macrolide susceptibility.

NLR, MLR and SIRI combine information from several circulating cell populations and have been investigated mainly as indicators of severity or prognosis. Higher NLR and SIRI have been associated with severe MP pneumonia and adverse outcomes (20–29). Evidence for MLR is less consistent: some studies found little diagnostic or prognostic value (10, 24), whereas others reported associations with clinical severity (26, 27). These studies generally used severe disease, complications or prognosis as their outcome, while the present study used 2063/2064 mutation status. The difference in outcome is important because a severity marker need not discriminate a resistance-associated mutation. None of the three indices differed between the mutation-status groups at diagnosis in this cohort. Their low *post-hoc* power means that small effects cannot be excluded, but the current results do not support using a single static inflammatory index to infer mutation status.

The longitudinal analysis provided a different perspective. Within the mutation-detected subgroup, MLR decreased from 0.32 (0.24–0.48) at the initial visit to 0.27 (0.19–0.37) at diagnosis. This pattern suggests that serial measurements may capture changes that are not apparent in a single cross-sectional comparison. Nevertheless, the result was confined to a subset with complete paired data and did not demonstrate a corresponding difference in the mutation-undetected group. Previous work has similarly shown that mutation status does not necessarily produce clear differences in routine blood indices (30). Thus, the paired finding is best interpreted as a temporal observation within mutation-detected patients rather than direct evidence that declining MLR identifies resistance.

Several processes could contribute to the observed MLR trajectory. The A2063G and A2064G mutations in domain V of the 23S rRNA gene reduce macrolide binding and may permit persistent infection despite initial treatment. Continued antigenic stimulation could promote mobilisation and recruitment of circulating monocytes to infected pulmonary tissue, where they differentiate into macrophages and participate in phagocytosis and cytokine production. At the same time, adaptive immune activation may alter circulating lymphocyte counts. Either process could lower the peripheral MLR. Disease severity and treatment modification may produce similar changes, however. Because pathogen burden, monocyte trafficking, lymphocyte activation and cytokines were not measured directly, this mechanism remains a plausible explanation rather than a pathway demonstrated by the present data.

The additional statistical analyses clarify the distinction between a group-level change and clinically useful discrimination. The Wilcoxon signed-rank test detected a change in the distribution of paired MLR values, whereas the bootstrap confidence interval for the mean paired difference included zero. These results are not necessarily contradictory because the two analyses address different features of the paired data, but together they indicate uncertainty in the magnitude and stability of the change. More importantly, MLR1 and MLR2 produced AUC values of only 0.508 and 0.523. Although MLR2 had a sensitivity of 85.9%, its specificity was 29.4%, implying a high false-positive rate. Statistical significance within a group therefore did not translate into useful individual-level classification, and MLR change should not be used as a stand-alone warning indicator.

The stratified and regression results point to clinical heterogeneity as another explanation. The MLR decline was significant among hospitalised patients but not among non-hospitalised patients, suggesting that the overall result was driven mainly by the inpatient subgroup. Hospitalisation may reflect greater severity, longer symptom duration, different treatment exposure or closer laboratory monitoring. Age and MLR at diagnosis were not independently associated with mutation-detected status. In the exploratory multivariable analysis, ΔMLR was also non-significant and its confidence interval was very wide. Complete paired measurements were insufficient in the mutation-undetected group to fit a reliable resistance-status model containing ΔMLR and the full covariate set. Taken together, these analyses weaken a resistance-specific interpretation and support viewing serial MLR as a descriptive measure of the clinical course.

The present findings define a narrower clinical role for MLR than initially proposed. A declining MLR should not replace 23S rRNA mutation testing, microbiological assessment or evaluation of disease severity, and it should not by itself trigger a change in antimicrobial therapy. Serial MLR may still provide supplementary information because it is inexpensive and routinely available. Its interpretation should be combined with fever duration, respiratory findings, imaging, hospitalisation status and other inflammatory markers. Future work should evaluate whether adding MLR change to a prespecified multivariable model improves discrimination beyond these established clinical variables. Such a model would require development and external validation in independent cohorts before clinical use.

Treatment exposure also needs to be considered when interpreting serial blood indices. Antimicrobial regimens are often modified according to age, severity and response to initial therapy, and these changes may alter pathogen burden and inflammation. Complete treatment histories were not available consistently in this retrospective cohort, precluding a reliable comparison between mutation-status groups. Tetracyclines or, in selected cases, respiratory fluoroquinolones may be considered for refractory or mutation-associated infection, while corticosteroids or intravenous immunoglobulin may be used when excessive inflammation is clinically evident. These decisions require individual assessment and should not be based on MLR alone. Prospective studies should record the initial regimen, timing of modification, adjunctive treatment and outcomes alongside serial haematological measurements.

This study has several strengths, including mutation-based grouping, paired measurements in a defined subgroup and the use of complementary robustness, discrimination and sensitivity analyses. Several limitations should also be considered. The retrospective, single-centre design and unequal group sizes limit generalisability. Respiratory nucleic-acid positivity did not distinguish lower respiratory infection from upper respiratory infection or carriage, and standardised severity scores were unavailable. Power was low for the secondary inflammatory indices, treatment information was incomplete and paired data in the mutation-undetected group were insufficient for a fully adjusted ΔMLR model. The ROC and multivariable analyses were exploratory and require external validation. Larger prospective cohorts with balanced mutation-status groups, standardised severity and treatment data, and paired sampling in both groups are needed to determine whether serial MLR adds information beyond established clinical indicators.

## 5 Conclusion

Static haematological parameters and inflammatory indices did not distinguish mutation-detected from mutation-undetected MP infection in this cohort. MLR decreased over time among mutation-detected patients with paired measurements, but ROC analysis showed poor discriminatory performance when the change was used alone. The decline was observed in hospitalised but not non-hospitalised patients, indicating that severity, treatment or other clinical factors may have contributed. Serial MLR may describe part of the inflammatory course, but the current evidence does not support its use as an independent warning or diagnostic indicator of macrolide resistance. Prospective studies with balanced mutation-status groups, standardised severity and treatment data, and paired sampling in both groups are required. Models combining MLR dynamics with clinical variables and other inflammatory markers should be evaluated before clinical application.

## Data Availability

The original contributions presented in the study are included in the article/Supplementary Material, further inquiries can be directed to the corresponding author.
