# Supplementary material for: Analysis of early haematological warning indicators for drug-resistant Mycoplasma pneumoniae infection
Source: Front Pediatr. 2026 Oct 21;14:1854263. doi: 10.3389/fped.2026.1854263 (PMC13731215; doi:10.3389/fped.2026.1854263)
Supplement: Supplementary file 1 [file Table1.doc]

**Supplementary Table S1. Post-hoc power analysis of inflammatory indices at diagnosis**

| **Index** | **Group** | **n** | **Mean rank** | **Z** | **p** | **Power** |
| --- | --- | --- | --- | --- | --- | --- |
| NLR | Mutation-detected | 134 | 79.94 | −0.643 | 0.521 | 0.11 |
|  | Mutation-undetected | 27 | 86.26 |  |  |  |
| MLR | Mutation-detected | 134 | 80.02 | −0.595 | 0.552 | 0.09 |
|  | Mutation-undetected | 27 | 85.87 |  |  |  |
| SIRI | Mutation-detected | 134 | 79.65 | −0.817 | 0.414 | 0.14 |
|  | Mutation-undetected | 27 | 87.69 |  |  |  |
| hs-CRP | Mutation-detected vs mutation-undetected | 161 | — | −1.010 | 0.313 | ≈0.17 |

Note: Effect size was estimated as r = Z/√N. Post-hoc power was calculated using a two-sided α level of 0.05.
