# Supplementary material for: Analysis of early haematological warning indicators for drug-resistant Mycoplasma pneumoniae infection
Source: Front Pediatr. 2026 Oct 21;14:1854263. doi: 10.3389/fped.2026.1854263 (PMC13731215; doi:10.3389/fped.2026.1854263)
Supplement: Supplementary file 2 [file Table2.doc]

**Supplementary Table S2. Logistic regression analysis of factors associated with mutation-detected status**

| **Variable** | **B** | **OR** | **95% CI** | **p** |
| --- | --- | --- | --- | --- |
| Age, years | −0.071 | 0.931 | 0.804–1.078 | 0.340 |
| MLR at diagnosis | 0.238 | 1.269 | 0.421–3.827 | 0.673 |
