# Supplementary material for: Analysis of early haematological warning indicators for drug-resistant Mycoplasma pneumoniae infection
Source: Front Pediatr. 2026 Oct 21;14:1854263. doi: 10.3389/fped.2026.1854263 (PMC13731215; doi:10.3389/fped.2026.1854263)
Supplement: Supplementary file 3 [file Table3.doc]

**Supplementary Table S3. Hospitalisation-stratified paired MLR analysis in the mutation-detected group**

| **MLR** | **Hospitalised (n=49)** | **Not hospitalised (n=15)** |
| --- | --- | --- |
| Initial visit | 0.35 (0.24–0.47) | 0.39 (0.23–0.52) |
| Diagnosis | 0.30 (0.19–0.34) | 0.37 (0.23–0.47) |
| Z | −2.92 | −0.70 |
| p | 0.004 | 0.480 |
