# Supplementary material for: Analysis of early haematological warning indicators for drug-resistant Mycoplasma pneumoniae infection
Source: Front Pediatr. 2026 Oct 21;14:1854263. doi: 10.3389/fped.2026.1854263 (PMC13731215; doi:10.3389/fped.2026.1854263)
Supplement: Supplementary file 4 [file Table4.doc]

**Supplementary Table S4. Exploratory multivariable logistic regression analysis**

| **Variable** | **B** | **OR** | **95% CI** | **p** |
| --- | --- | --- | --- | --- |
| Sex, male/female | −1.117 | 0.327 | 0.091–1.176 | 0.087 |
| Age, years | 0.098 | 1.103 | 0.888–1.370 | 0.374 |
| Hospitalisation, yes/no | −0.511 | 0.600 | 0.162–2.215 | 0.443 |
| ΔMLR | 2.143 | 8.525 | 0.360–201.918 | 0.180 |
| ΔCRP | −0.013 | 0.988 | 0.960–1.016 | 0.388 |
| Fever duration before diagnosis | 0.161 | 1.174 | 0.933–1.478 | 0.171 |
